# Evolving *Escherichia coli* Host Strains for Efficient Deuterium Labeling of Recombinant Proteins Using Sodium Pyruvate-*d*_3_

**DOI:** 10.3390/ijms22189678

**Published:** 2021-09-07

**Authors:** Vinardas Kelpšas, Anna Leung, Claes von Wachenfeldt

**Affiliations:** 1 Department of Biology, Lund University, Sölvegatan 35, SE-223 62 Lund, Sweden; vinardas.kelpsas@biol.lu.se; 2 Scientific Activities Division, European Spallation Source ERIC, P.O. Box 176, SE-221 00 Lund, Sweden; Anna.Leung@ess.eu

**Keywords:** deuteration, neutron crystallography, isotope labelling, adaptive experimental evolution

## Abstract

Labeling of proteins with deuterium (2H) is often necessary for structural biology techniques, such as neutron crystallography, NMR spectroscopy, and small-angle neutron scattering. Perdeuteration in which all protium (1H) atoms are replaced by deuterium is a costly process. Typically, expression hosts are grown in a defined medium with heavy water as the solvent, which is supplemented with a deuterated carbon source. *Escherichia coli*, which is the most widely used host for recombinant protein production, can utilize several compounds as a carbon source. Glycerol-*d*_8_ is often used as a carbon source for deuterium labelling due to its lower cost compered to glucose-*d*_7_. In order to expand available options for recombinant protein deuteration, we investigated the possibility of producing a deuterated carbon source in-house. *E. coli* can utilize pyruvate as a carbon source and pyruvate-*d*_3_ can be made by a relatively simple procedure. To circumvent the very poor growth of *E. coli* in minimal media with pyruvate as sole carbon source, adaptive laboratory evolution for strain improvement was applied. *E. coli* strains with enhanced growth in minimal pyruvate medium was subjected to whole genome sequencing and the genetic changes were revealed. One of the evolved strains was adapted for the widely used T7 RNA polymerase overexpression systems. Using the improved strain *E. coli* DAP1(DE3) and in-house produced deuterated carbon source (pyruvic acid-*d*_4_ and sodium pyruvate-*d*_3_), we produce deuterated (>90%) triose-phosphate isomerase, at quantities sufficient enough for large volume crystal production and subsequent analysis by neutron crystallography.

## 1. Introduction

Deuterium (D or ^2^H) is a naturally occurring stable isotope of hydrogen (H, ^1^H, or protium) [1]. D and H have the largest mass ratio among stable isotopes of the same element giving rise to differences in physico-chemical properties that can be exploited in various techniques to gain information on structural aspects of biomolecules. For example, protein labelling with deuterium is used in small-angle neutron scattering (SANS), neutron reflectometry, nuclear magnetic resonance (NMR) spectroscopy, and neutron crystallography. Deuterium labelling in SANS can be used for contrast variation [2], while it provides increased sensitivity and simplified spectra for protein NMR [3]. Neutron crystallography is a complementary technique to X-ray crystallography that provides structural information about hydrogen positions in proteins [4,5]. As approximately one-half of all atoms in a protein are H atoms, information on their 3D positions are of importance to reveal details of hydrogen bonding and protonation state. Such information is crucial to understanding substrate recognition and catalysis at the atomic level. X-rays interact with electrons, whereas neutrons interact with atomic nuclei. For X-rays, the scattering length is a linear function of the atomic number. Thus, light elements such as hydrogen scatter scarcely and are in general very poorly resolved in X-ray structures of proteins, unless they are determined at ultrahigh-resolution (<0.8 Å). The nuclear scattering lengths of C, D, N, O, and S are roughly comparable in magnitude [4]. However, the most abundant hydrogen isotope, protium, has a negative scattering length that leads to signal cancellation of positive scattering neighboring atoms, and a large incoherent cross-section giving rise to a significant background in collected neutron crystallography data [6]. Therefore, when H is exchanged for D, the quality of the diffraction data drastically improves. In order to reach close to complete deuteration of a protein (perdeuteration), the organism producing the protein of interest is grown in a heavy water (D_2_O)-based defined growth medium supplemented with a deuterated carbon source. Current price for D_2_O and deuterated carbon source (typically glucose-*d*_7_ or glycerol-*d*_8_) is high, thus perdeuteration in a larger scale is not always a realizable option. Moreover, growth of microorganisms in deuterated growth medium is slow, and may result in poor biomass yield and require long adaptation procedures [7,8]. 

*Escherichia coli* is the most common expression host used for recombinant protein production. *E. coli* can utilize a wide variety of sugars, organic acids, and other organic compounds as sole carbon source [9]. The three-carbon compound pyruvate (CH_3_COCOO^−^) is a terminal product of glycolysis and a starting substrate for the tricarboxylic acid cycle. *E. coli* can use pyruvate as sole carbon and energy source [10]. Pyruvate is metabolized via the gluconeogenesis pathway to generate glucose and by the tricarboxylic acid cycle to produce amino acids. During anaerobic growth, it is a precursor for the generation of fermentation products like, acetate, ethanol, and L-lactate. A straightforward procedure for the production of sodium pyruvate-*d*_3_ has been reported previously [11]. In this procedure, the methyl protons of pyruvic acid are exchanged by incubation with D_2_O, after which a mild base (sodium bicarbonate) is used to convert pyruvic acid-*d*_4_ to the sodium salt, which facilitates subsequent isolation of the product and subsequent recrystallization. By using this procedure, sodium pyruvate-*d*_3_ could in principle be made in-house and used as a deuterated carbon source for growth of *E. coli* to produce perdeuterated recombinant proteins at a reduced cost. However, the very poor growth of *E. coli* in minimal media with pyruvate has made this approach less attractive. Here, we applied adaptive laboratory evolution (ALE) with the aim of improving growth of *E. coli* in a defined minimal medium with pyruvate as carbon source. ALE involves the continued growth of a bacterial population under an appropriate selective pressure [12]. Fitter mutants are selected from random mutations occurring mainly during DNA replication. By continuous growth for many generations, the frequency of a particular mutant in the population will increase in proportion to its fitness. ALE has been used to successfully improve microbial strains for various industrial applications, such as enhanced substrate utilization, improved growth rate and resistance to toxic compounds [13,14,15]. By using this approach, *E. coli* strains with greatly improved growth rate in deuterated minimal medium with pyruvate as carbon source were obtained and the genetic modifications of such strains is reported. We also report a facile procedure for in-house sodium pyruvate-*d*_3_ production for use in subsequent production of perdeuterated recombinant soluble and membrane-proteins. 

## 2. Results and Discussion

### 2.1. Adaptation of E. coli for Growth in Minimal Pyruvate Medium

Growth of *E. coli* is typically very slow in deuterated minimal media [16]. A reduced growth rate not only increases the time required for protein production, but can be also be problematic when unstable or volatile media components are used. For example, pyruvate tends to slowly dimerize in solution to form parapyruvate, suggesting that long culture incubations would lead to loss of the available carbon source [17]. Thus, the first objective of this work was to obtain a strain with improved growth rate in a defined deuterated minimal medium with unlabeled pyruvate as carbon source (referred here to as D-M9). We used ALE, which is a well-established approach for directed evolution, to find variants with improved competitive fitness for growth in D-M9. The *E. coli* K-12 strain MG1655 was used as the parental strain for the ALE experiment. Three lineages (named P1, P2, and P3) were subjected to approximately 280 generations of growth in D-M9, comprising a total of 49 subculturings. At the end point of the adaptive evolution, one isolated clone from each lineage was tested for growth in D-M9 media. The P1 and P3 isolates showed the largest improvement in growth compared to the parental strain, while the P2 isolate had only a modest increase in growth rate (Table 1 and Figure S1). The P3 (P3.49.1) isolate had a growth rate of 0.235 ± 0.0004 h^−1^, more than fourfold that of the parental strain.

### 2.2. Observed Mutations in the Evolved Strains

To find causal mutations that underlie the improved strain performance, chromosomal DNA was extracted from two isolated clones of each lineage at the end point of the adaptive evolution and used for whole-genome resequencing. The observed mutations are reported in Table 2. The P3 lineage had accumulated the highest number of mutations. In addition to resequencing the genomes from pure isolates, we also subjected the population (mixture of clones) to sequencing. We assume that mutations of high frequency are fixed in the population and thus provide adaptation to growth media. Interestingly, there were no mutations directly related to pyruvate metabolism except for one mutation in the *ldhA* gene encoding D-lactate dehydrogenase of the P3.49.1 strain. The missense mutation changes a conserved glycine at position 78 for alanine. D-lactate dehydrogenase carries out the reduction of pyruvate to D-lactate under anaerobic conditions, but it is also present at aerobic conditions [18]. It has been reported that substitution of the conserved glycine (G78) with alanine in *Sporolactobacillus inulinus* D-LDH (Lactate Dehydrogenase) results in less than 5% of wild-type enzyme activity [19]. This suggests that in strain P3.49.1, D-LDH is inactivated and may result in an altered metabolic flux that is beneficial to growth on pyruvate.

Strains P1.49.1, P1.49.2, and P2.49.1 had a duplication (a repetition of 81 nucleotides) in *rhlB*. This gene encodes RhlB, which is an ATP-*d*ependent RNA helicase that mediates unwinding of double-stranded RNA. Since this mutation was not detected in all of the sequenced lineages, it will not be further discussed.

All analyzed strains had mutations in the *rpoS* gene encoding the major alternative sigma factor, σ^S^ (or sigma 38), which regulates the general stress response and stationary phase gene expression in *E. coli* [20]. The observed amino acid substitutions in σ^S^ are in close proximity to each other suggesting that they have a related effect on σ^S^ function. In the strains P1.49.1 and P3.49.1, the substitutions in σ^S^ are present in a conserved sequence motif (called motif 2 or region 2) and in P2.49.1 in motif 3. The function of motif 3 is poorly understood [21]. Motif 2 is also present in the other σ^70^ family of sigma factors RpoD and RpoH [22]. RpoD is the primary sigma factor responsible for gene regulation during the exponential growth phase [23]. RpoH is involved in the heat shock response [24]. This motif is of importance for binding to RNA polymerase (RNAP) via interaction with the β’-subunit (RpoC). Substitutions similar to those we observed here in σ^S^ (strains P1.49.1 and P3.49.1) have been made in RpoD (σ^70^). The variant RpoD show a 15-fold reduced affinity to RNAP compared to the wild-type protein [25]. This suggests that the observed substitutions in the evolved strains could result in reduced affinity of σ^S^ to RNAP. Thus, modulating σ^S^ activity appear to be beneficial in deuterated conditions. The levels of σ^S^ are low in exponential growth but increase several fold upon entry into stationary phase. Many genes in *E. coli* are positively controlled by σ^S^ and several genes are negatively controlled. Thus, altered σ^S^ activity may affect expression of hundreds of genes and provide a selective advantage during growth of deuterated media. A functional σ^S^, albeit with reduced activity, seems important as it would be more likely to obtain null mutations completely lacking σ^S^ activity.

All analyzed strains had mutations in the *ilvG* pseudogene. The *ilvG* gene in *E. coli* K-12 strains contain a frameshift near the middle of the gene. The observed mutations restore the reading frame of *ilvG*. The restored *ilvG* gene encodes acetohydroxyacid synthase II (AHAS II), an enzyme that together with IlvM, catalyzes the biosynthesis of α-aceto-α-hydroxybutyrate for the isoleucine pathway and of α-acetolactate for the valine pathway. Lack of AHAS II is suggested to induce recurrent isoleucine starvation. Thus, rewiring isoleucine metabolism is likely to provide a selective advantage for growth in D-M9. Interestingly, no mutations in the *rpoC* (encoding RNA polymerase β′-subunit) or the *rpoB* (encoding RNA polymerase β-subunit) genes were detected. In previous reported ALE experiments, mutations in these genes are most frequently observed, which can be explained by that such mutations lead to large scale transcriptional reprogramming [14,26,27].

### 2.3. Deuteration of Pyruvate

**Figure 1:**
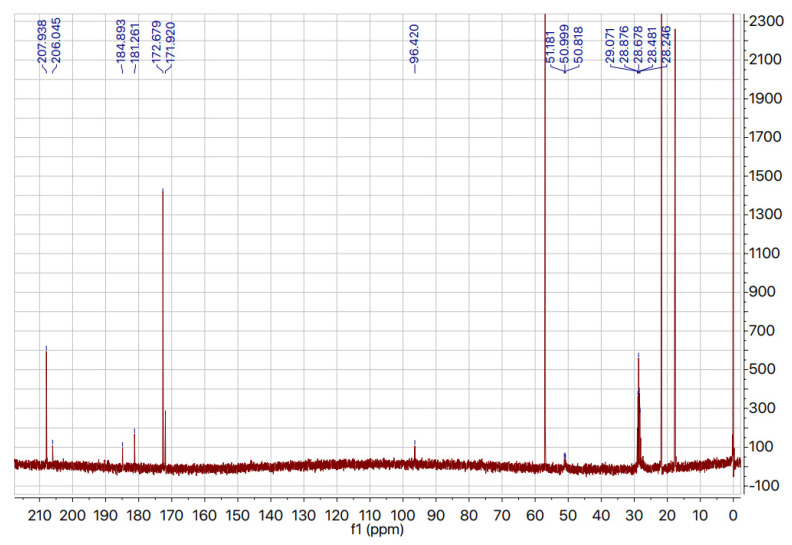
^13^C NMR spectrum of sodium pyruvate-*d*_3_ in D2O with 1% (*w*/*w*) 3-(trimethylsilyl)-1-propanesulfonic acid, sodium salt (DSS) (reference)(x-axis: chemical shift (ppm); y-axis: intensity of signal). Peaks at 181.261 and 96.420 ppm indicate presence of pyruvate hydrate. Peaks at 181.9, 184.9, 206.0, and 50.0 ppm indicate possible presence of parapyruvate (dimer). Peaks between 29.1 and 28.2 ppm indicate C*d*_3_ signal of sodium pyruvate-*d*_3_.

**Figure 2:**
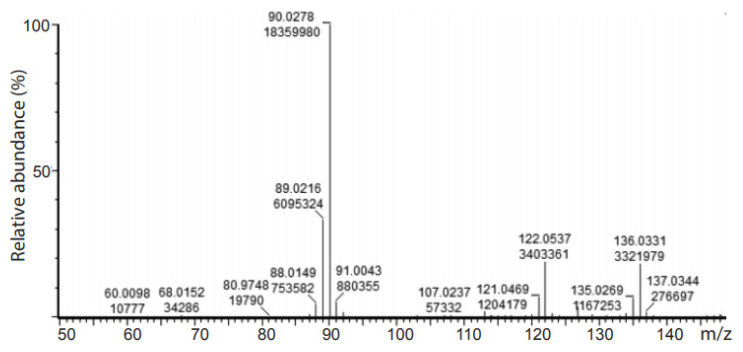
High resolution mass spectrum of the deuterated sodium pyruvate: *m*/*z* calculated for C_3_D_3_O_3_– [M–H]–: 90.03; found: 90.03; calculated for C_3_D_2_HO_3_– [M–H]–: 89.02; found: 89.02; calculated for C_3_DH_2_O_3_– [M–H]–: 88.02; found: 88.01.

Several studies have reported H/D exchange of the methyl group of pyruvic acid [11,28,29,30]. Robson and co-workers report the use of strongly basic conditions (2.5 mM NaOD, pH 13.0) in a minimal deuterium-based medium to exchange pyruvate methyl protons for deuterons. Thirty minutes after addition of NaOD, the solution was neutralized and used directly as a growth medium. The in situ nature of this exchange may seem practical, but other reports claim that the use of a strong base such as NaOH (or NaOD) causes pyruvate to dimerize to form parapyruvate [11]. It has been shown that in solutions with a pH of 9 or above, more than 50% of pyruvate dimerizes to form parapyruvate at room temperature [17]. For this reason, we chose not to use strong basic conditions for the H/D exchange. The method of Shchepin and co-workers [11] was chosen because it included subsequent sodium salt formation, which simplifies purification, and increased the amount of purified product obtained. In an effort to reduce the cost associated with the exchange reaction, we halved the ratio of D_2_O to pyruvic acid in this report (112.5 mL D_2_O/g of pyruvic acid compared to 225 mL D_2_O/g pyruvic acid). Under these conditions, the extent of deuteration was nevertheless almost as high as was previously reported [11]. The product was isolated as the sodium salt and recrystallized using the conditions of Shchepin and co-workers [11]. The ^13^C NMR spectrum of the sodium salt was recorded in D_2_O with the sodium salt of 3-(trimethylsilyl)-1-propanesulfonic acid, as an internal calibration standard. Under these conditions, pyruvate-*d*_3_ and a small amount of pyruvate-*d*_3_ hydrate could be observed, in addition to a small amount of another related species, tentatively assigned as either pyruvic acid-*d*_3_ or parapyruvate-*d*_5_ (Figure 1). It was difficult to identify this species from a comparison with the literature spectrum for the unlabeled sample, because deuteration is known to affect chemical shift, and because the spectra were obtained in different solvents (a mixture of H_2_O and D_2_O in one instance, and in 100% D_2_O in the other) [17]. The deuteration level of sodium pyruvate was calculated using the isotope distribution of the different isotopologues in the mass spectrum, with a calculated deuteration level reaching 90% (the ratio of C_3_D_3_O_3_^–^ to C_3_D_2_HO_3_^−^ to C_3_DH_2_O_3_^−^ was 1:0.33:0.04) (Figure 2).

### 2.4. Production of Perdeuterated Proteins in the Pyruvate Adapted Strain

Currently, one of the most widely used bacterial protein-producing systems uses the bacteriophage T7 polymerase that recognizes the T7 promoter [31]. To enable protein production by T7 RNA polymerase promoter-based plasmids in the evolved strain P3.49.1, it was lysogenized with the λ bacteriophage DE3 that carries the T7 gene 1 encoding the T7 RNA polymerase under control of the isopropyl *β*-D-1-thiogalactopyranoside (IPTG) inducible *lac*UV5 promoter. The resulting strain was named DAP1(DE3) (Deuterium Adapted Pyruvate strain 1) and was used for recombinant protein production. Three test proteins were produced; a superfolding derivative of green fluorescent protein (sfGFP), *Leishmania mexicana* triosephosphate isomerase (TIM), which is a key enzyme in glycolysis that catalyzes the interconversion of glyceraldehyde 3-phosphate and dihydroxyacetone phosphate and the outer membrane protein F (OmpF) of *E. coli*. TIM is a very well-studied enzyme, but some details on its catalytic mechanism remains to be resolved [32]. Here, we used a site-*d*irected mutant (E97Q) of a residue that is situated close to the active site to enable future neutron crystallographic studies. The E97Q TIM variant was produced in DAP1(DE3) grown in DD-M9 supplemented with recrystallized sodium pyruvate-*d*_3_ as the sole carbon source. After protein production and purification, we obtained approximately 18 mg deuterated TIM variant E97Q from cells grown in 1 l medium. The deuteration level was estimated by intact mass analysis by mass spectrometry to approximately 97% (Table 3). As a comparison, using DD-M9 but with glycerol-*d*_8_ as sole carbon source typically yields 25 to 37 mg of purified deuterated TIM [33]. Thus, using *E. coli* DAPI(DE3), the deuterated TIM variant E97Q could be produced in quantities and levels of D incorporation appropriate for large volume crystal growth and subsequent analysis by neutron crystallography.

**Figure 3:**
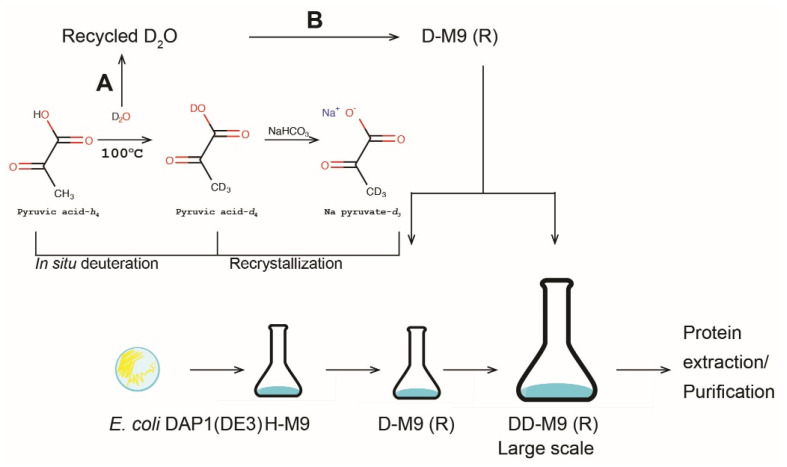
Framework of production of sodium pyruvate-*d*_3_, and deuteration of recombinant proteins. Deuterated pyruvic acid is produced by boiling pyruvic acid in D_2_O. This solution can be immediately used as base for growth medium or sodium pyruvate-*d*_3_ can be recrystallized. The heavy water (D_2_O) used in pyruvate deuteration could be used for subsequent labelling procedures (A) or used as solvent for growth media (B).

**Figure 4:**
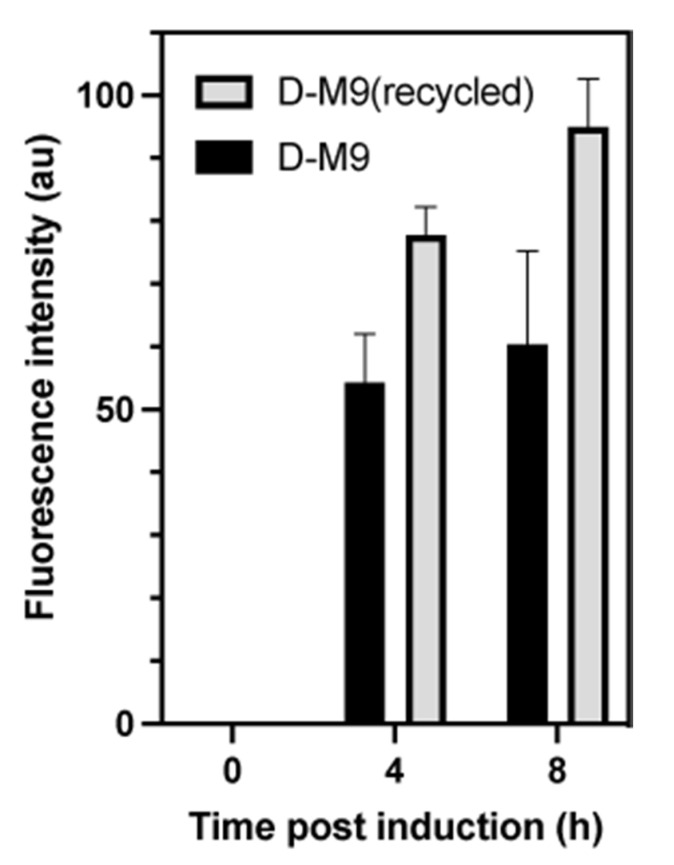
Protein production using *E. coli* DAP1(DE3). Absolute fluorescence intensities (arbitrary units) in cells harvested at different time points after inducing expression of the gene encoding sfGFP. *E. coli* DAP1(DE3) was grown at 37 °C in D-M9 and D-M9 prepared with recycled D_2_O. Protein expression was induced at an OD_600_ of ~1. Each histogram represents three biological replicates. Error bars represents the standard error of the mean.

The method employed to prepare sodium pyruvate-*d*_3_ required 932 mL of heavy water per 5 g of purified pyruvate-*d*_3_. The heavy water was recovered from the reaction mixture by distillation and a small amount (50–100 mL) was utilized for re-crystallization of the product. The remaining heavy water can be recycled by distillation in a rotary evaporator and may be used for other purposes as illustrated in Figure 3. Recycling by this procedure typically yields 96−98% D_2_O [34]. We tested if the recycled heavy water can be used to prepare growth media for deuterated protein production. By using sfGFP as a reporter of protein production, we did not observe reduced sfGFP levels in media prepared from recycled heavy water (Figure 4). Interestingly, recycled D-M9 yielded higher amount of sfGFP, which could be due to that the recycled heavy water contained a higher fraction of light water compared to the regular D-M9 medium.

An alternative procedure to conserve heavy water would be to produce perdeuterated pyruvic acid in situ. To test this procedure, we omitted the isolation and recrystallization steps from the methods to prepare pyruvic acid-*d*_4_ and used this solution as base for preparation of the growth medium. When TIM was produced in DAP1(DE3) from a 0.5 L culture, 14 mg of 98% deuterated TIM (Table 4) was obtained.

**Figure 5:**
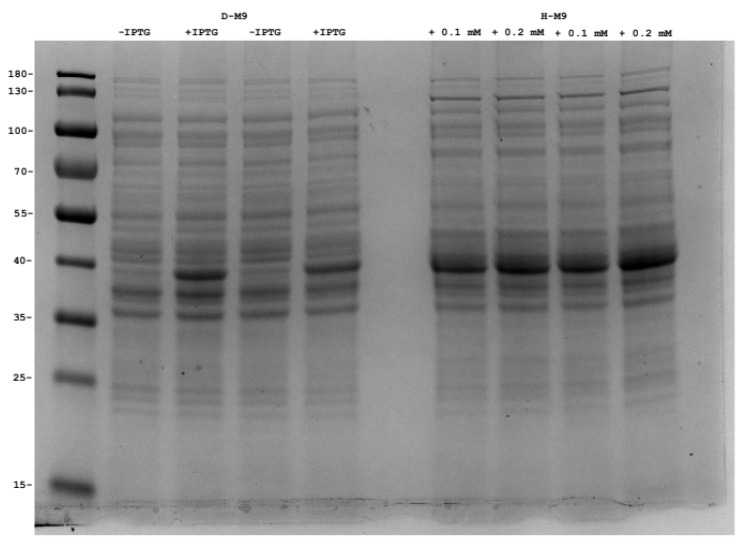
SDS-PAGE analysis of OmpF production. Whole cell fraction of *E. coli* DAP1(DE3) (Δ*ompF*) expressing *ompF* cultured cultured at 37 °C in D-M9 and H-M9. Two different concentrations (0.1 or 0.2 mM) of the inducer IPTG was used in H-M9 media. For the D-M9 media 0.1 mM IPTG was used. The culture grown in H-M9 was induced at an OD600 of 0.5 and the D-M9 culture at 1.5. Cells were harvested 8 (H-M9) and 15 (D-M9) hours after induction. Each condition was done with two biological replicates and the samples were loaded at cultured volume equivalents. The right lane was loaded with a molecular size marker ladder (kDa). Recombinant OmpF has a predicted mass of 39.5 kDa.

Production of membrane proteins usually have more inherent problems compared to expression of cytoplasmic proteins [35]. In order to show that the evolved strain DAP1(DE3) can be used to perdeuterate not only soluble proteins, we produced the outer membrane protein OmpF of *E. coli*. To facilitate future studies with OmpF variants, the native *ompF* gene was deleted and replaced with a kanamycin resistance cassette. The *ompF* gene was expressed from a plasmid and produced OmpF with an N-terminal extension consisting of His_6_, spacer, and a protease-cleavage sequence. Small-scale test expression was done in deuterated and non-*d*euterated M9 medium. SDS-PAGE analysis indicate that similar levels of OmpF is obtained in the D-M9 medium with pyruvate-*h*_3_ compared to M9 medium with pyruvate-*h*_3_. (Figure 5). Taken together, these experiments show that *E. coli* DA1(DE3) is suitable for production of recombinant perdeuterated proteins using in-house produced sodium pyruvate-*d*_3_.

## 3. Materials and Methods

### 3.1. Strains and Growth Conditions

*Escherichia coli* K-12 MG1655 [36] was used as the parental strain. Bacteria were revived from glycerol stocks by streaking onto Lysogeny broth (LB) with 1.5% agar (LA) and incubation at 37 °C. Media were supplemented when needed with 50 µg/mL kanamycin. LB, NaCl 10 g/L, Difco yeast extract 5 g/L, Difco tryptone 10 g/L, made up with H_2_O, pH 7.6, was used as a rich medium. The M9 minimal medium (referred to as H-M9) consisted of Na_2_HPO_4_ • 2H_2_O, 42.7 mM, KH_2_PO_4_ 22 mM, NaCl 8.6 mM, NH_4_Cl 107 mM, MgSO_4_ • 7 H_2_O 1 mM, CaCl_2_ 0.1 mM, thiamine HCl, 2 mg/L, FeCl_3_ • 6H_2_O 0.018 mM, sodium pyruvate (Sigma-Aldrich, Stockholm, Sweden) 5–10 g/L as sole carbon source. To induce expression of recombinant protein 0.5 mM IPTG was added to the growth media. When deuterated media were prepared, all solutions were made freshly in heavy water (D_2_O, 99.8% D-atom, Sigma-Aldrich, Stockholm, Sweden y) and then filtered with 0.22 µm sterile filter (VWR—vacuum filtration unit, VWR Stockholm, Sweden). It is important to note that deuterated media should not be autoclaved. The deuterated media that contained non-labelled sodium pyruvate is referred to as D-M9. Media with labelled sodium pyruvate-*d*_3_ is referred to as DD-M9. All salts used in D-M9 and DD-M9 were the same as in M9, but instead of Na_2_HPO_4_ • 2H_2_O, anhydrous Na_2_HPO_4_ was used. An appropriate amount of sodium pyruvate was dissolved in H_2_O or D_2_O just before the start of growth experiments. For perdeuterated protein production, the stock solution of IPTG was prepared in heavy water. Plates and liquid cultures were incubated at 37 °C.

### 3.2. Sodium Pyruvate-d_3_ Production

Pyruvate-*d*_3_ was made using a modification of a previously published procedure, [11]. Pyruvic acid (6.00 g, 68.1 mmol) and D_2_O (675 mL) were heated to refluxing temperature of 100 °C for 5.5 h. The mixture was allowed to cool to room temperature and sodium bicarbonate (5.43 g, 64.6 mmol) was added slowly with constant stirring. The solvent was removed under reduced pressure and the resulting solid was recrystallized using a mixture of the recovered D_2_O from the reaction and absolute ethanol (in a ratio of 1:3.75) to afford sodium pyruvate-*d*_3_ as a white solid (3.62 g, 50% yield, 90% D by mass spectrometry (MS)). ^13^C NMR (D_2_O with 1% (*w*/*w*) 3-(trimethylsilyl)-1-propanesulfonic acid, sodium salt, 100 MHz) δ 28.7 (m), 172.7, 207.9. HRMS (ESI–) *m*/*z* calculated for C_3_D_3_O_3_ [M–Na]^–^ as 90.03; found: 90.03. Deuteration: 90% by MS: isotope distribution: *d*_1_ 1.0%, *d*_2_ 16.2%, *d*_3_ 72.7%.

### 3.3. In Situ Deuteration of Pyruvic Acid

A total of 3.5 g of pyruvic acid was mixed with 500 mL D_2_O and heated to refluxing temperature of 100 °C for 5.5 h, and mixture was prepared as outlined above. Mixture was allowed to cool to room temperature. All M9 medium components were dissolved in this solution and pD was adjusted to 7.64 by adding NaOD (99.5% D) (Sigma Aldrich, Stockholm, Sweden). This solution was immediately used to set up bacterial growth cultures.

### 3.4. Growth Experiments

Overnight bacterial cultures were set-up by inoculating a few colonies from an LA plate of the appropriate strain into 25 mL H-M9, and incubated over-night at 37 °C, 200 rpm. The over-night cultures were diluted approximately 20 times to an optical density at 600 nm (OD_600_) of 0.1. This was done by harvesting an appropriate amount of culture by centrifugation (8 min at 8000× *g* at 20 °C), after which the supernatant was carefully removed and the pellet was suspended in 25 mL D-M9 medium. The cultures were then incubated at 37 °C. Growth rate experiments were performed by measuring the OD_600_ of duplicate cultures over several time points at cell densities between 0.05 and 2. The specific growth rate μ was calculated as the slope of the linear best-fit line through a plot of ln (OD_600_) versus time (hours). The generation time (or doubling), *t_d_*, is equal to ln2/μ.

### 3.5. ALE and Strain Isolation

The parental strain *E. coli* MG1655 (K-12) was streaked on an LA plate and incubated overnight at 37 °C. The following day, a few colonies were used to inoculate 25 mL H-M9 in a 250 mL baffled Erlenmeyer flask that was incubated at 37 °C, 200 rpm until the culture reached an OD_600_ of 3. The culture was centrifuged for 8 min at 8000× *g*, the supernatant was removed and the cell pellet suspended in 15 mL D-M9 (10 g/L sodium pyruvate-*h*_3_), and divided into equal parts (5 mL) in three TPP TubeSpin bioreactor 50 mL tubes, at 37 °C inclined at a 45° angle with shaking at 200 rpm. The cultures are referred to as three separate population lineages (P1, P2, and P3). The batch cultures were grown until they reached approximately OD_600_ = 3, then the exact OD_600_ was recorded and used to estimate the number of generations passed from day one. Next, each lineage was used to inoculate 5 mL fresh D-M9. Cultures were manually transferred to fresh medium. Cultures before the third subculturing started at an OD_600_ of 0.1 (1:30 dilution), after this time point, they started at an OD_600_ of 0.05 (1:60 dilution). Subculturing was carried out every 48 h for three subculturings, and after that for every day. For every three subcultures, 660 µL of culture was mixed with 340 µL of 86% glycerol and stored at −82 °C. Samples of each culture was regularly inspected for contamination by streaking for single colonies on LA plates. After ALE, single colonies (clones) were isolated by repeatedly (three times) streaking and incubating on H-M9 agar (supplemented with 10 mg/mL pyruvate). Finally, single colonies were used to streak a bacterial lawn on LA plates, which was later used to prepare a glycerol stock for long-term freezer storage. 

### 3.6. Whole-Genome DNA Sequencing

Illumina sequencing technology was used for whole genome shot-gun sequencing. Genomic RNA-free DNA was purified using the DNeasy Blood and Tissue Kit (Qiagen, Hilden, Germany) according to the manufacturer’s instructions. Illumina sequencing was done at GATC Eurofins Genomics (Konstanz, Germany). Genome sequencer Illumina HiSeq was used with 2 × 150 bp paired-end read output. Approximately 10 million reads per sample were mapped using CLC genomics workbench (Version 11, Qiagen, Hilden, Germany) to the reference genome sequence of *E. coli* MG1655 (Genbank entry code: U00096.3), obtained from the NCBI genome repository. Observed differences were compared to the parental strain from our laboratory stocked MG1655 strain, which was previously sequenced [16].

### 3.7. Lysogenization

In order to test recombinant protein production capabilities, the gene (gene 1) for T7 RNA polymerase was introduced into strains of interest by lysogenizing with the lambda phage DE3 using the λDE3 Lysogenization Kit (Novagen, Darmstadt, Germany) as previously described [16].

### 3.8. Deletion of ompF

Deletion of and *ompF* was done using the λ-Red recombinase-mediated gene deletion method [37]. A PCR product harboring 50 bp end sequences homologous to *ompF* and a chloramphenicol resistance marker was amplified with plasmid pKD3 as template and primers *ompF*_up (5′-ATTGACGGCAGTGGCAGGTGTCATAAAAAAAACCATGAGGGTAATAAATAGTGTAGGCTGGAGCTGCTTC-3′) and *ompF*_down (5′-AAACAGGACCAAAGTCCTGTTTTTTCGGCATTTAACAAAGAGGTGTGCTAATGGGAATTAGCCATGGTCC-3′). The PCR product was transformed into the appropriate *E. coli* strain harboring the λ-Red recombinase expression plasmid pKD46, and subsequently transformants were selected on media plates containing chloramphenicol. Deletion of *ompF* was verified by amplifying the appropriate chromosomal region using primer pairs and *ompF1* (5′-CACTTTCACGGTAGCGAAAC-3′)/*ompF2* (5′-CATGACGAGGTTCCATTATGG-3′), and confirming by Sanger DNA sequencing (Eurofins).

### 3.9. Perdeuteration of TIM

For protein perdeuteration, *E. coli* DAP1(DE3) was transformed with plasmid pET24a(+)_Lm_TIM (E97Q). Cultures were inoculated and set-up as outlined under “growth experiments”. D-M9 culture was used as inoculum for DD-M9 culture, which was incubated at 37 °C. At an OD_600_ of 1, IPTG to a final concentration of 0.5 mM was added. Cultures were further incubated for 15 h. Biomass was harvested by centrifugation, the supernatant was carefully removed, and then frozen at −80 °C, until further use. Protein purification was carried as described previously [33]. Intact mass of purified protein was performed on MALDI TOF/TOF mass spectrometer (Bruker Autoflex Speed TOF/TOF MALDI-MS) and compared to hydrogenated protein, to determine the degree of deuteration.

### 3.10. GFP and OmpF Expression

*E. coli* DAP1(DE3) was transformed with plasmid pETM14_sfGFP [16]. Cultures were set up as outlined under “growth experiments” in D-M9. Cultures were set-up in D-M9 and D-M9 (R) (recycled heavy water from pyruvate labelling) in triplicates. At an OD_600_ of 1 IPTG to a final concentration of 0.5 mM was added. At different time points, OD_600_ was recorded and 1 mL of culture was collected, centrifuged at 8000× *g* for 8 min at 4 °C. The supernatant was carefully removed, and the cell pellet was suspended in 0.5 mL cold 50 mM Tris, 100 mM NaCl, 10 mM EDTA buffer (pH 8.0). Samples were then diluted 100-fold in 100 mM NaCl, 100 mM sodium-phosphate buffer (pH 7.5) sfGFP fluorescence (excitation 485 nm, emission 510 nm) was measured. Fluorescence was measured using an RF-5301 Spectrofluorophotometer (Shimadzu, Kyoto, Japan) and fluorescence intensity was recorded after subtraction of background fluorescence for the buffer used. *E. coli* DAP1(DE3) was transformed with plasmid pET-24a(+)_ompF that contains the *E. coli ompF* gene modified with a coding sequence of a hexa-histidine tag followed by a TEV endopeptidase cleavage recognition sequence placed between the OmpF signal sequence and native OmpF.

## 4. Conclusions

In this study, we show that high deuterium content (~90%) sodium pyruvate-*d*_3_ can be prepared in a conventional chemistry laboratory. In combination, an ALE generated faster growing *E. coli* strain and sodium pyruvate-*d*_3_, TIM variant E97Q was produced and perdeuterated at above 90% with a yield, which according to our experience, is enough for production of crystals for one complete neutron diffraction dataset [33]. Moreover, recycled heavy water obtained from the production of sodium pyruvate-*d*_3_ is not toxic to *E. coli* and does not appear to reduce production of recombinant protein. Using in-house prepared pyruvate-*d*_3_ does not reduce costs compared to using media with glycerol-*d*_8_ due to recombinant protein yields being lower with pyruvate-*d*_3_. However, if the recycled heavy water from the production of sodium pyruvate-*d*_3_ is used to prepare growth media, protein perdeuteration can be done at a reduced cost comparable to using glycerol-*d*_8_. Moreover, we show that in situ preparation of pyruvic acid-*d*_4_, reduces solvent waste, with no apparent reduction in yield or deuteration level of recombinant protein. By using in situ preparation of pyruvic acid-*d*_4_, the cost of the growth medium for deuteration is largely equal to the cost of D_2_O.

## Figures and Tables

**Table 1:** Growth rates of parental and evolved *E. coli* strains.

Strain	Growth Rate (h^−1^) ^a^	Generation Time (h)
MG1655	0.056 ± 0.0002	12.4
P1.49.1	0.199 ± 0.0095	3.5
P2.49.1	0.103 ± 0.0002	6.7
P3.49.1	0.235 ± 0.004	2.9

^a^ Cells were grown in D-M9 with 5 mg/mL of sodium pyruvate-*h*_3._

**Table 2:** Mutations identified in selected clones and populations after adaptation in deuterated minimal medium with sodium pyruvate-*h*_3_.

Position ^a^	Gene	Strain									Coding Region Change ^c^	Amino Acid Change ^d^	Description
		P1.49			P2.49			P3.49					
		1	2	Frequency (%) ^b^	1	2	Frequency (%) ^b^	1	2	Frequency (%) ^b^			
1316251	**Non coding**									99.2	A > G	NA	
1330830	**Non coding**										C > T	NA	
1356902	*sapA*										209T > G	Leu70Arg	Putative periplasmic binding protein
1397277	**Non coding**										G > T	NA	
1442611	*ldhA*										233G > C	Gly78Ala	D-lactate dehydrogenase
1861387	**Non coding**										G > A	NA	
1918327	*yebT*										818A > G	Asp273Gly	Intermembrane transport protein
2339829	*ubiG*										263T > G	Phe88Cys	Ubiquinol-8 biosynthesis protein
2423134	*yfcI*										406T > G	Trp136Gly	Recombination-promoting nuclease
2867121	*rpoS*						97.2				431C > T	Thr144Ile	Sigma S
2867169	*rpoS*			98.3							383T > A	Ile128Asn	Sigma S
2867182	*rpoS*									99.8	370A > T	Asn124Tyr	Sigma S
3856831	*yidl*										417C > T	NA	Putative DNA-binding transcriptional regulator
3951538	*ilvG*			66.2							979del	Gln327fs	Isoleucine biosynthesis
3951543	*ilvG*						44.8			99.7	984del	*328fs	Isoleucine biosynthesis
3965550	*rhlB*			87.7			35.0				81dup	Gly28Fs	ATP-*d*ependent RNA helicase RhlB
4033620	*trkH*									65.7	476A > T	Gln159Leu	K^+^ transporter

^a^ Nucleotide position in the reference sequence Genbank entry: U00096.3. ^b^ The frequency of the indicated mutations was determined in the population at the 49th subculturing from the respective lineage. ^c^ Deletion (del), duplication (dup). ^d^ Non-sense mutation (*). Frame shift (fs).

**Table 3:** Intact mass MS analysis of proteins isolated from cells grown in DD-M9 (recrystallized pyruvate-*d*_3_).

Sample ^a^	Calculated Mass (Da)	Observed Mass (Da)
D-TIM	28,791	28,772
H-TIM	27,264	27,285
Mass difference	1527	1487

^a^ TIM E97Q (H-TIM) and deuterated TIM E97Q (D-TIM).

**Table 4:** Intact mass MS analysis of proteins isolated from cells grown in DD-M9 (in situ perdeuterated pyruvic acid).

Sample ^a^	Calculated Mass (Da)	Observed Mass (Da)
D-TIM	28,791	28,793
H-TIM	27,264	27,285
Mass difference	1527	1508

^a^ TIM E97Q (H-TIM) and deuterated TIM E97Q (D-TIM).

## Data Availability

Data is contained within the article.

## Supplementary Materials

The following are available online at https://www.mdpi.com/article/10.3390/ijms22189678/s1.

## Abbreviations

AHAS II	Acetohydroxyacid synthase II
ALE	Adaptive laboratory evolution
EDTA	Ethylenediaminetetraacetic acid
IPTG	Isopropyl *β*-D-1-thiogalactopyranoside
NMR	Nuclear magnetic resonance
OmpF	Outer membrane protein F
SANS	Small-angle neutron scattering
TIM	Triose-phosphate isomerase from *Leishmania mexicana*
H-M9	M9 minimal medium with pyruvate as carbon source.
D-M9	M9 minimal medium made up with D_2_O and pyruvate as carbon source.
DD-M9	M9 minimal medium made up with D_2_O and deuterated pyruvate as carbon source.

## References

[1] Berglund M., Wieser M.E. (2011). Isotopic compositions of the elements 2009 (IUPAC Technical Report). Pure Appl. Chem..

[2] Zaccai N.R., Sandlin C.W., Hoopes J.T., Curtis J.E., Fleming P.J., Fleming K.G., Krueger S. (2016). Deuterium labeling together with contrast variation small-angle neutron scattering suggests how Skp captures and releases unfolded outer membrane proteins. Methods Enzymol..

[3] Yu H. (1999). Extending the size limit of protein nuclear magnetic resonance. Proc. Natl. Acad. Sci. USA.

[4] Oksanen E., Chen J.C., Fisher S.Z. (2017). Neutron crystallography for the study of hydrogen bonds in macromolecules. Molecules.

[5] Helliwell J.R. (2020). Fundamentals of neutron crystallography in structural biology. Methods Enzymol..

[6] Blakeley M. (2009). Neutron macromolecular crystallography. Crystallogr. Rev..

[7] Crespi H.L., Conard S.M., Uphaus R.A., Katz J.J., Conrad S.M. (1960). Cultivation of microorganisms in heavy water. Ann. N. Y. Acad. Sci..

[8] Haertlein M., Moulin M., Devos J.M., Laux V., Dunne O., Forsyth V.T. (2016). Biomolecular deuteration for neutron structural biology and dynamics. Methods Enzymol..

[9] Aidelberg G., Towbin B.D., Rothschild D., Dekel E., Bren A., Alon U. (2014). Hierarchy of non-glucose sugars in *Escherichia coli*. BMC Syst. Biol..

[10] Cooper R.A., Kornberg H.L. (1967). The direct synthesis of phospho enol pyruvate from pyruvate by *Escherichia coli*. Proc. R. Soc. Lond. Ser. B Boil. Sci..

[11] Shchepin R.V., Coffey A.M., Waddell K.W., Chekmenev E.Y. (2014). Parahydrogen Induced polarization of 1-13C-phospholactate-*d* 2 for Biomedical imaging with >30,000,000-fold NMR signal enhancement in water. Anal. Chem..

[12] Sandberg T.E., Salazar M.J., Weng L.L., Palsson B.O., Feist A.M. (2019). The emergence of adaptive laboratory evolution as an efficient tool for biological discovery and industrial biotechnology. Metab. Eng..

[13] Atsumi S., Wu T., Machado I.M.P., Huang W., Chen P.-Y., Pellegrini M., Liao J. (2010). Evolution, genomic analysis, and reconstruction of isobutanol tolerance in *Escherichia coli*. Mol. Syst. Biol..

[14] Conrad T.M., Frazier M., Joyce A.R., Cho B.-K., Knight E.M., Lewis N., Landick R., Palsson B.O. (2010). RNA polymerase mutants found through adaptive evolution reprogram *Escherichia coli* for optimal growth in minimal media. Proc. Natl. Acad. Sci. USA.

[15] Minty J.J., A Lesnefsky A., Lin F., Chen Y., A Zaroff T., Veloso A.B., Xie B., A McConnell C., Ward R.J., Schwartz D.R. (2011). Evolution combined with genomic study elucidates genetic bases of isobutanol tolerance in *Escherichia coli*. Microb. Cell Factories.

[16] Kelpsas V., von Wachenfeldt C. (2019). Strain improvement of *Escherichia coli* K-12 for recombinant production of deuterated proteins. Sci. Rep..

[17] Margolis S.A., Coxon B. (1986). Identification and quantitation of the impurities in sodium pyruvate. Anal. Chem..

[18] Jiang G.R., Nikolova S., Clark D.P. (2001). Regulation of the *ldhA* gene, encoding the fermentative lactate dehydrogenase of *Escherichia coli*. Microbiology.

[19] Zhu L., Xu X., Wang L., Dong H., Yu B. (2015). The D-lactate dehydrogenase from *Sporolactobacillus inulinus* also possessing reversible deamination activity. PLoS ONE.

[20] Landini P., Egli T., Wolf J., Lacour S. (2014). sigma S, a major player in the response to environmental stresses in *Escherichia coli*: Role, regulation and mechanisms of promoter recognition. Environ. Microbiol. Rep..

[21] Campbell E.A., Muzzin O., Chlenov M., Sun J.L., Olson C., Weinman O., Trester-Zedlitz M.L., Darst S.A. (2002). Structure of the bacterial RNA polymerase promoter specificity σ subunit. Mol. Cell.

[22] Wösten M. (1998). Eubacterial sigma-factors. FEMS Microbiol. Rev..

[23] Maciąg A., Peano C., Pietrelli A., Egli T., De Bellis G., Landini P. (2011). In vitro transcription profiling of the σ S subunit of bacterial RNA polymerase: Re-*d*efinition of the σS regulon and identification of σS-specific promoter sequence elements. Nucleic Acids Res..

[24] Straus D.B., Walter W.A., Gross C.A. (1987). The heat shock response of *E. coli* is regulated by changes in the concentration of σ32. Nature.

[25] Sharp M.M., Chan C.L., Lu C.Z., Marr M.T., Nechaev S., Merritt E.W., Severinov K., Roberts J.W., Gross C.A. (1999). The interface of sigma with core RNA polymerase is extensive, conserved, and functionally specialized. Genes Dev..

[26] Tenaillon O., Rodríguez-Verdugo A., Gaut R.L., McDonald P., Bennett A.F., Long A.D., Gaut B.S. (2012). The molecular diversity of adaptive convergence. Science.

[27] Deatherage D.E., Kepner J.L., Bennett A.F., Lenski R.E., Barrick J.E. (2017). Specificity of genome evolution in experimental populations of *Escherichia coli* evolved at different temperatures. Proc. Natl. Acad. Sci. USA.

[28] White R.H. (1978). Stable isotope studies on the biosynthesis of the thiazole moiety of thiamin in *Escherichia coli*. Biochemistry.

[29] Guzman M.I., Colussi A.J., Hoffmann M.R. (2006). Photogeneration of distant radical pairs in aqueous pyruvic acid glasses. J. Phys. Chem. A.

[30] Robson S.A., Takeuchi K., Boeszoermenyi A., Coote P.W., Dubey A., Hyberts S., Wagner G., Arthanari H. (2018). Mixed pyruvate labeling enables backbone resonance assignment of large proteins using a single experiment. Nat. Commun..

[31] Studier F.W., Moffatt B.A. (1986). Use of bacteriophage T7 RNA polymerase to direct selective high-level expression of cloned genes. J. Mol. Biol..

[32] Kelpsas V., Caldararu O., Blakeley M.P., Coquelle N., Wierenga R.K., Ryde U., von Wachenfeldt C., Oksanen E. (2021). Neutron structures of *Leishmania mexicana* triosephosphate isomerase in complex with reaction-intermediate mimics shed light on the proton-shuttling steps. IUCrJ.

[33] Kelpšas V., Lafumat B., Blakeley M.P., Coquelle N., Oksanen E., von Wachenfeldt C. (2019). Perdeuteration, large crystal growth and neutron data collection of *Leishmania mexicana* triose-phosphate isomerase E65Q variant. Acta Crystallogr. Sect. F Struct. Biol. Cryst. Commun..

[34] Koruza K., Lafumat B., Végvári Á., Knecht W., Fisher S. (2018). Deuteration of human carbonic anhydrase for neutron crystallography: Cell culture media, protein thermostability, and crystallization behavior. Arch. Biochem. Biophys..

[35] Carpenter E.P., Beis K., Cameron A.D., Iwata S. (2008). Overcoming the challenges of membrane protein crystallography. Curr. Opin. Struct. Biol..

[36] Blattner F.R., Plunkett G., Bloch C.A., Perna N.T., Burland V., Riley M., Collado-Vides J., Glasner J.D., Rode C.K., Mayhew G.F. (1997). The complete genome sequence of *Escherichia coli* K-12. Science.

[37] Datsenko K.A., Wanner B.L. (2000). One-step inactivation of chromosomal genes in *Escherichia coli* K-12 using PCR products. Proc. Natl. Acad. Sci. USA.

