# Supplementary figures and images for: Evolving Escherichia coli Host Strains for Efficient Deuterium Labeling of Recombinant Proteins Using Sodium Pyruvate-d3
Source: Int J Mol Sci. 2021 Sep 7;22(18):9678. doi: 10.3390/ijms22189678 (PMC8465070; doi:10.3390/ijms22189678)

*E. coli* growth in D-M9

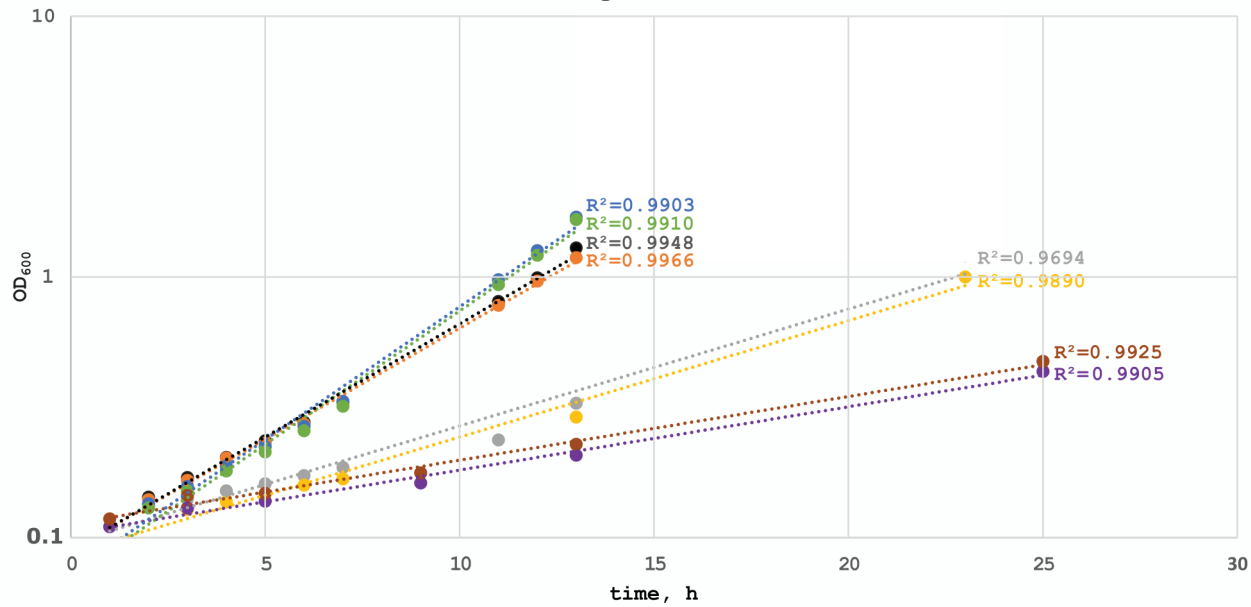

| Symbol | Strain  | Growth rate h <sup>-1</sup> |
|--------|---------|-----------------------------|
| ● ●    | P1.49.1 | 0.199 ± 0.0095              |
| ● ●    | P2.49.1 | 0.103 ± 0.0002              |
| ● ●    | P3.49.1 | 0.235 ± 0.0004              |
| ● ●    | MG1655  | 0.056 ± 0.0002              |

Supplement: Supplementary file 1 [file ijms-22-09678-s001.zip › ijms-1343756-supplementary.pdf]
